# Quercetin Decreases Claudin-2 Expression Mediated by Up-Regulation of microRNA miR-16 in Lung Adenocarcinoma A549 Cells

**DOI:** 10.3390/nu7064578

**Published:** 2015-06-08

**Authors:** Hiroyuki Sonoki, Tomonari Sato, Satoshi Endo, Toshiyuki Matsunaga, Masahiko Yamaguchi, Yasuhiro Yamazaki, Junko Sugatani, Akira Ikari

**Affiliations:** 1 Laboratory of Biochemistry, Department of Biopharmaceutical Sciences, Gifu Pharmaceutical University, Gifu 501-1196, Japan; E-Mails: 116023@gifu-pu.ac.jp (H.S.); sendo@gifu-pu.ac.jp (S.E.); matsunagat@gifu-pu.ac.jp (T.M.); 2 School of Pharmaceutical Sciences, University of Shizuoka, Shizuoka 422-8526, Japan; E-Mails: final-detective-l@ezweb.ne.jp (T.S.); masahiko-y@u-shizuoka-ken.ac.jp (M.Y.); y-yamaza@u-shizuoka-ken.ac.jp (Y.Y.); sugatani@u-shizuoka-ken.ac.jp (J.S.)

**Keywords:** lung adenocarcinoma, claudin-2, miR-16, quercetin

## Abstract

Claudin-2 is highly expressed in human lung adenocarcinoma tissues and cells. Knockdown of claudin-2 decreases cell proliferation and migration. Claudin-2 may be a novel target for lung adenocarcinoma. However, there are no physiologically active substances of foods which decrease claudin-2 expression. We here found that quercetin, a flavonoid present in fruits and vegetables, time- and concentration-dependently decreases claudin-2 expression in lung adenocarcinoma A549 cells. In the present study, we examined what regulatory mechanism is involved in the decrease in claudin-2 expression by quercetin. Claudin-2 expression was decreased by LY-294002, a phosphatidylinositol 3-kinase (PI3-K) inhibitor, and U0126, a MEK inhibitor. These drugs inhibited the phosphorylation of Akt and ERK1/2, which are downstream targets of PI3-K and MEK, respectively. In contrast, quercetin did not inhibit the phosphorylation. Both LY-294002 and U0126 inhibited promoter activity of claudin-2, but quercetin did not. The stability of claudin-2 mRNA was decreased by quercetin. Quercetin increased the expression of microRNA miR-16. An inhibitor of miR-16 rescued quercetin-induced decrease in the claudin-2 expression. These results suggest that quercetin decreases claudin-2 expression mediated by up-regulation of miR-16 expression and instability of claudin-2 mRNA in lung adenocarcinoma cells.

## 1. Introduction

Flavonoids present in fruits, vegetables, and plants have profound pharmacological properties, and a daily intake of them is associated with a decreased risk of cancer [1]. Quercetin is one of the major flavonoids found in human diet. Quercetin shows apoptotic and anti-proliferative effects in various human cancer cells derived from gastrointestinal tract [2], breast [3], prostate [4], and lung [5]. Quercetin induces apoptosis mediated via reduction of Bcl-2 family protein, release of cytochrome c, and activation of caspases [6,7]. In contrast, the underlying molecular mechanism of anti-proliferation has not been fully understood.

Tight junctions (TJs) seal adjacent cells of epithelia in a narrow band at the apical pole of the lateral membrane. TJs regulate the flux of ions and solutes through the paracellular pathway, cell proliferation, and differentiation [8,9,10]. Claudins are integral membrane proteins in the formation of TJs and comprise a large family of 27 subtypes [11,12]. The functions of TJs may be regulated by the combination of claudin subtypes. Dysregulation of claudins expression has been shown in various tumor tissues [13]. The expression of claudin-1 and -7 is up-regulated and that of claudin-2 is down-regulated in human lung adenocarcinoma. The improvement of the expression level of these claudins decreases cell proliferation or migration in NSCLC cells [14,15,16]. Cell proliferation and migration are regulated by several intracellular signaling pathways including a MEK/ERK and phosphatidylinositol 3-kinase (PI3-K)/Akt. Therefore, it is important to clarify the relationship between these signaling pathways and the expression of claudins.

MicroRNAs (miRNAs) are small and non-coding RNA molecules (21–23 nt) bind to the complementary recognition sequences in the 3′-untranslated region (3′-UTR) of target mRNAs and act as a negative regulator of gene expression either by blocking mRNA translation or RNA interference [17,18]. miRNAs play essential roles in a variety of biological and pathological processes including cell proliferation, migration, and apoptosis. The target and function of miRNA are different in each tissue. The specific miRNA acts as either oncogenes or tumor suppressors, depending on the target gene.

miRNA microarray studies reveal that various miRNAs are aberrantly expressed in lung cancer [19]. miR-16 expression is down-regulated in human non-small cell lung cancer (NSCLC) tissue samples compared with normal tissues. The ectopic expression of miR-16 decreases hepatoma-derived growth factor, a potential oncogene, and inhibits cell growth, migration, and invasion in NSCLC cells [20], indicating that miR-16 functions as a negative regulator of cell cycle progression. Furthermore, miR-16 has manifold cellular functions in other tissues. miR-16 inhibits the transcriptional activity of nuclear factor-kappaB and Slug, resulting in suppression of epithelial-mesenchymal transition in human glioma [21]. miR-16 expression is inversely correlated with Bcl-2 expression, which induces apoptosis in leukemic cells [22]. The correlation of miRNAs and claudins has been reported in several cancer cells. Claudin-1 expression is suppressed by miR-155 in colorectal [23] and ovarian cancer cells [24]. In contrast, the expression is up-regulated by miR-198 in hepatocellular carcinoma cells [25]. Claudin-18 expression is suppressed by miR-1303 in gastric cancer cells [26]. However, there is no report that claudin-2 expression is regulated by miRNA.

In the present study, we found that quercetin decreases claudin-2 expression by decreasing the stability of its mRNA in A549 cells. Quercetin increased miR-16 expression and an inhibitor of miR-16 rescued quercetin-induced decrease in claudin-2. Our results indicate that quercetin may decrease claudin-2 expression through increasing miR-16 expression in lung adenocarcinoma cells.

## 2. Experimental Section

### 2.1. Materials

Anti-claudin-1 and claudin-2 antibodies were obtained from Zymed Laboratories (South San Francisco, CA, USA). Anti-β-actin, c-Fos, and phosphorylated-c-Fos (p-c-Fos) antibodies were from Santa Cruz Biotechnology (Santa Cruz, CA, USA). Quercetin and U0126 were from Wako Pure Chemical Industries (Osaka, Japan). LY-294002 was from BIOMOL Research Laboratories (Plymouth Meeting, PA, USA). Cordycepin was from Focus Biomolecules (Hamburg, Germany). U0126, LY-294002, quercetin and cordycepin were dissolved in dimethylsulfoxide (DMSO). All other reagents were of the highest grade of purity available.

### 2.2. Cell Culture and Transfection

The human lung adenocarcinoma A549 cell line was obtained from the RIKEN BRC through the National Bio-Resource Project of the MEXT, Japan. Cells were grown in Dulbecco’s modified Eagle’s medium (DMEM, Sigma-Aldrich) supplemented with 5% fetal calf serum (FCS, HyClone, Logan, UT, USA), 0.07 mg/mL penicillin-G potassium, and 0.14 mg/mL streptomycin sulfate in a 5% CO_2_ atmosphere at 37 °C. The experiments were done in subconfluent culture condition (about 70%–80% confluent), because the expression of claudin-2 decreased in 100% confluent condition [16]. The cells were treated with vehicle DMSO (control), quercetin (0.5–100 μM), U0126 (10 μM), or LY-294002 (10 μM) for 24 h in FCS-free DMEM. Negative control or miR-16 inhibitor (anti-miR-16 specific antisense oligonucleotide) was transfected into A549 cells with Lipofectamine 2000 as recommended by the manufacturer. They then underwent 48 h of transfection.

### 2.3. SDS-Polyacrylamide Gel Electrophoresis (SDS-PAGE) and Immunoblotting

The preparation of cytoplasmic extracts was performed as described previously [16]. Samples were applied to SDS-PAGE and blotted onto a PVDF membrane. The membrane was then incubated with each primary antibody (1:1000 dilution) at 4 °C for 16 h, followed by a peroxidase-conjugated secondary antibody (1:5000 dilution) at room temperature for 1 h. Finally, the blots were incubated in Pierce Western Blotting Substrate (Thermo Fisher Scientific, Waltham, MA, USA) and exposed to film, or incubated in ECL Prime Western Blotting Detection System (GE Healthcare UK Ltd., Buckinghamshire, UK) and scanned with a C-DiGit Blot Scanner (LI-COR Biotechnology, Lincoln, NE, USA). Band density was quantified with ImageJ software (National Institute of Health, Bethesda, MD, USA).

### 2.4. RNA Isolation and Quantitative RT-PCR

Total RNA was isolated from A549 cells using TRI reagent (Sigma-Aldrich, St Louis, MO, USA). Reverse transcription was carried out with ReverTra Ace qPCR RT Kit (Toyobo Life Science, Osaka, Japan). Quantitative real time PCR was performed using FastStart Universal SYBR Green Master (Roche Applied Science, Mannheim, Germany). The primers used to PCR are listed in Supplementary Table 1. The threshold cycle (Ct) for each PCR product was calculated with the instrument’s software, and Ct values obtained for claudin-1 and -2 were normalized by subtracting the Ct values obtained for β-actin. In the experiments of miRNAs, reverse transcription was carried out with Mir-X miRNA First-Strand Synthesis Kit (Takara, Osaka, Japan). Quantitative real time PCR was performed using the specific primers for miRNAs (forward) and mRQ 3′ primer (reverse). The primers are listed in Supplementary Table S1. The Ct values obtained for miRNAs were normalized by subtracting the Ct values obtained for U6 snRNA as recommended by the manufacturer’s instructions. The resulting ∆Ct values were then used to calculate the relative change in mRNA expression as a ratio (R) according to the equation R = 2^−(∆Ct (treatment) − ∆Ct (control))^.

### 2.5. 3′-Rapid Amplification of cDNA Ends (RACE) PCR

3′-RACE PCR was carried out using 3′-Full RACE Core Set (Takara). Revers Transcription was carried out using Oligo dT-3 sites adaptor primer. PCR reaction was carried out using 3 sites adaptor primer and claudin-2 specific primer (5′-ATTTGAGATTGGAGAGGCTCTTTAC-3′) under the following conditions: denaturation at 94 °C for 0.5 min, annealing at 54 °C for 0.5 min, and extension at 72 °C for 0.5 min; these steps were repeated 30 cycles. The PCR products were visualized with ethidium bromide after electrophoretic separation on a 2% agarose gel.

### 2.6. Luciferase Reporter Assay

Using the reporter plasmid containing fragment of −1031/+37 of human claudin-2, luciferase reporter assay was carried out as described previously [27]. Relative promoter activity was represented as the fold-increase compared to the promoterless pGL4.10 vector.

### 2.7. Statistics

Results are presented as means ± S.E.M. Differences between groups were analyzed with a one-way analysis of variance, and corrections for multiple comparison were made using Tukey’s multiple comparison test. Comparisons between two groups were made using Student’s *t* test. Statics were performed using KaleidaGraph version 4.5.1 software (Synergy Software, PA, USA). Significant differences were assumed at *p* < 0.05.

## 3. Results

### 3.1. Decrease in Claudin-2 Expression by Quercetin

**Figure 1:**
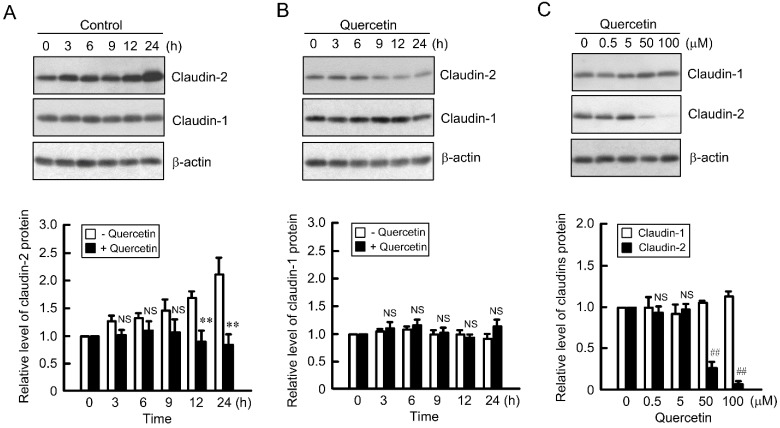
Decrease in claudin-2 expression by quercetin. (**A** and **B**) A549 cells were incubated in the absence (−) and presence (+) of 50 μM quercetin for the periods indicated. Cytoplasmic fractions were immunoblotted with anti-claudin-2, claudin-1, or β-actin antibody. The expression of claudin-1 and claudin-2 was represented relative to the values in 0 h; (**C**) Cells were incubated with quercetin at the indicated concentrations for 24 h. Cytoplasmic fractions were immunoblotted with anti-claudin-1, claudin-2, or β-actin antibody. The expression of claudin-1 and claudin-2 was represented relative to the values in 0 μM. *n* = 3–4. ** *p* < 0.01 significantly different from −quercetin. ^##^ *p* < 0.01 significantly different from 0 μM. NS, not significantly different.

Claudin-2 is highly expressed in human lung adenocarcinoma tissues and A549 cells compared with that in the normal lung tissue [28]. The expression level of claudin-2 protein in the cytoplasmic fraction time-dependently increased in A549 cells, which was inhibited by quercetin (Figure 1A). In contrast, claudin-1 expression was unchanged by the treatments with quercetin (Figure 1B). Furthermore, quercetin decreased the expression level of claudin-2 protein in a dose-dependent manner (Figure 1C). These results indicate that quercetin decreases claudin-2 expression without affecting claudin-1 expression in A549 cells.

### 3.2. Effect of Quercetin on the Phosphorylation of Akt and ERK1/2

**Figure 2:**
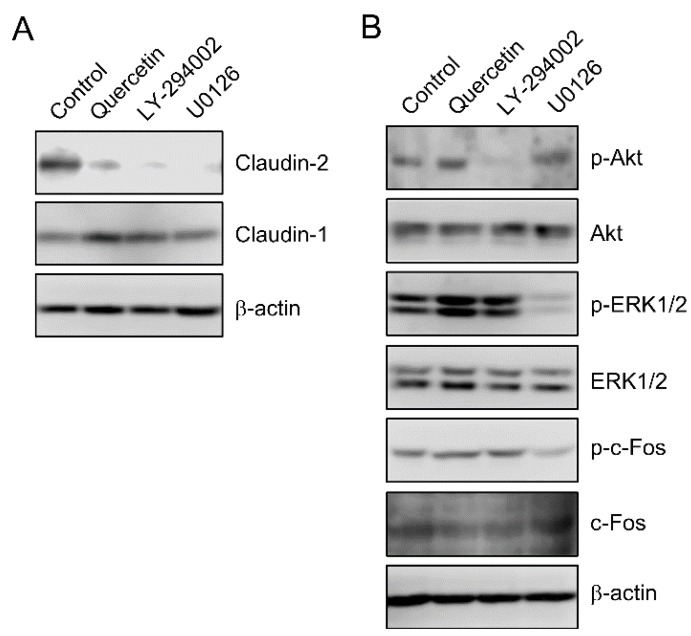
Effect of quercetin on the phosphorylation of ERK1/2 and Akt. (**A**) A549 cells were incubated in the absence (control) and presence of 50 μM quercetin, 10 μM LY-294002, or 10 μM U0126 for 24 h. Cytoplasmic fractions were immunoblotted with anti-claudin-1, claudin-2, or β-actin antibody; (**B**) Cells were incubated in the absence (control) and presence of 50 μM quercetin, 10 μM LY-294002, or 10 μM U0126 for 30 min. Cytoplasmic fractions were immunoblotted with anti-p-Akt, Akt, p-ERK1/2, ERK1/2, p-c-Fos, c-Fos, or β-actin antibody.

The expression level of claudin-2 protein was decreased by LY-294002, a PI3-K inhibitor, and U0126, a MEK inhibitor (Figure 2A). LY-294002 and U0126 inhibited the phosphorylation of Akt and ERK1/2, respectively (Figure 2B). In contrast, quercetin had no effect on the phosphorylation of Akt and ERK1/2. Claudin-2 expression is decreased by down-regulation of p-c-Fos, a down-stream target of ERK1/2 [27]. However, quercetin did not decrease p-c-Fos level. These results indicate that ERK1/2 and Akt are not involved in the decrease in claudin-2 expression by quercetin.

### 3.3. Inhibition of Promoter Activity by Quercetin

**Figure 3:**
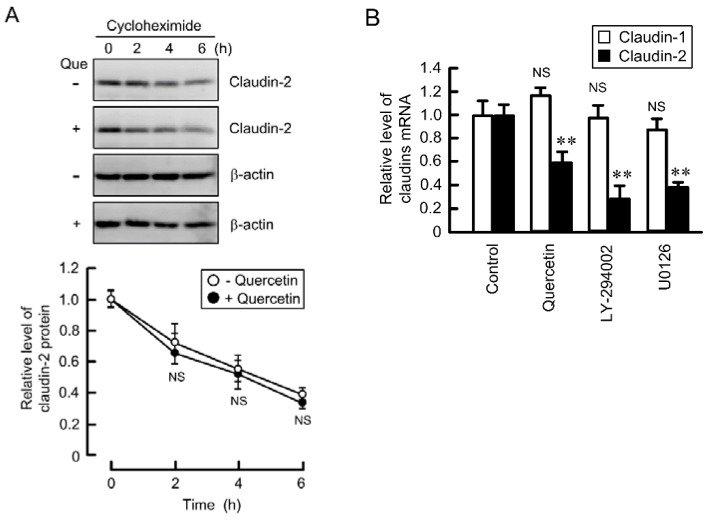
Effect of quercetin on the protein stability and mRNA level of claudin-2. (**A**) A549 cells were treated with 10 μM cycloheximide in the absence (−) and presence (+) of 50 μM quercetin for the period indicated. Cytoplasmic fractions were immunoblotted with anti-claudin-2 or β-actin antibody. The expression of claudin-2 was represented relative to the values in 0 h; (**B**) Cells were incubated in the absence (control) and presence of 50 μM quercetin, 10 μM LY-294002, or 10 μM U0126 for 6 h. The expression level of mRNA was quantified by real-time PCR using primers for claudin-1, claudin-2, and β-actin, and was represented relative to values at control. *n* = 3–4. ** *p* < 0.01 significantly different from control. NS, not significantly different.

**Figure 4:**
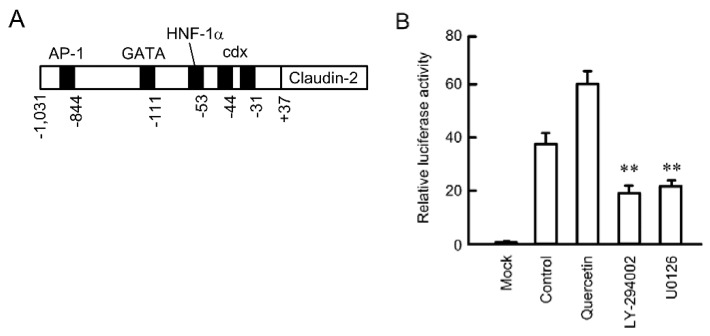
Effect of quercetin on the reporter activity of claudin-2. (**A**) Schematic drawing of transcriptional binding sites in human claudin-2 promoter; (**B**) A549 cells were co-transfected with luciferase pGL4.10 vector and pRL-TK vector. After 24 h of transfection, the cells were incubated in the absence and presence of 50 μM quercetin, 10 μM LY-294002, or 10 μM U0126 for 24 h. The relative promoter activity of claudin-2 was represented as the fold induction compared to that of the promoter-less pGL4.10 vector (mock). *n* = 4. ** *p* < 0.01 significantly different from control.

The expression level of protein is affected by the level of mRNA and/or the stability of protein. The expression level of claudin-2 protein was time-dependently decreased in the presence of cycloheximide, a translational inhibitor (Figure 3A). Quercetin did not change the rate of decrease, indicating that quercetin did not affect the stability of claudin-2 protein. The expression level of claudin-2 mRNA was significantly decreased by quercetin, LY-294002, and U0126 (Figure 3B). These results are consistent with those of Western blotting. Furthermore, quercetin decreased the expression level of claudin-2 mRNA in lung adenocarcinoma cell lines such as PC-3 and ABC-1 (Supplementary Figure S1). The transcriptional activity of claudin-2 is up-regulated by STAT3 in Madin Darby canine kidney (MDCK) cells [29], GATA4 in intestinal HIEC-6 cells [30], and cdx1, cdx2 and hepatocyte nuclear factor-1 in intestinal Caco-2 cells [31]. These transcriptional biding sites were schematically shown in Figure 4A. We recently reported that an EGFR/MEK/ERK/c-Fos pathway is involved in the up-regulation of promoter activity of claudin-2 in A549 cells [27]. Both U0126 and LY-294002 inhibited luciferase promoter activity (Figure 4B). In contrast, quercetin enhanced rather than inhibited it.

### 3.4. Decrease in the Stability of Claudin-2 mRNA by Quercetin

**Figure 5:**
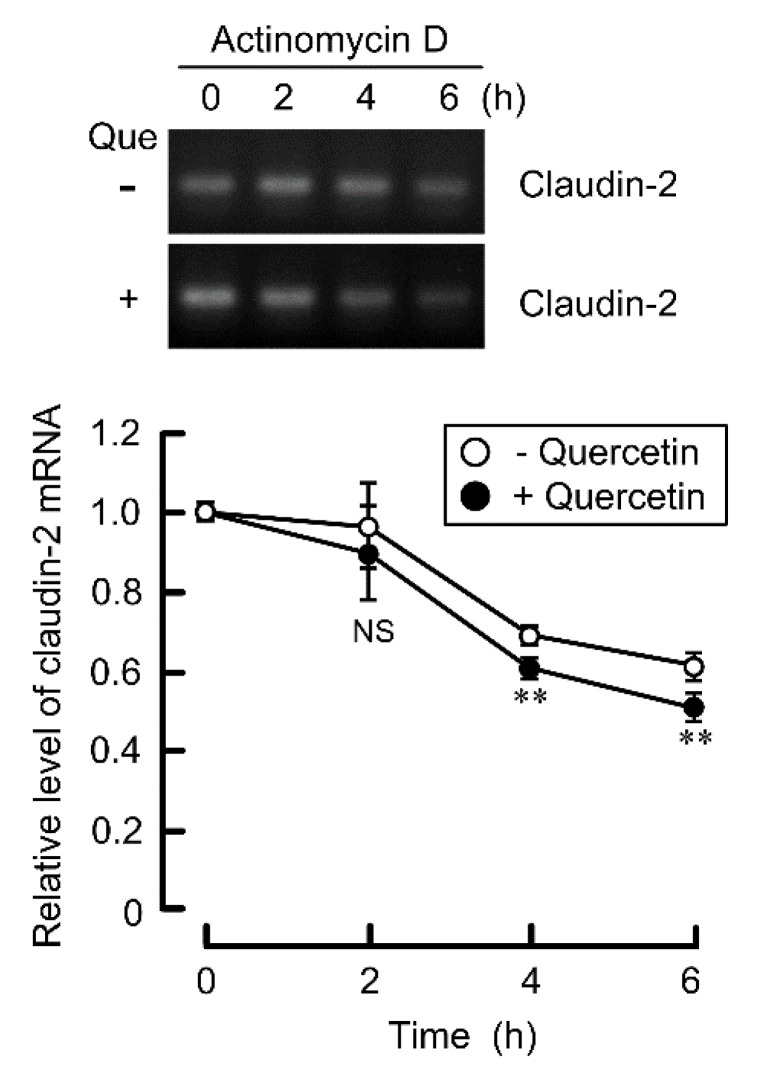
Decrease in the stability of claudin-2 mRNA by quercetin. A549 cells were treated with 10 μM actinomycin D in the absence and presence of 50 μM quercetin for the periods indicated. After isolation of total RNA, semi-quantitative RT-PCR (upper images) and quantitative real time PCR (lower graph) were performed using specific primers for claudin-2. *n* = 3. ** *p* < 0.01 is significantly different from control.

To clarify whether quercetin decreases stability of claudin-2 mRNA, we examined the expression of claudin-2 in the presence of actinomycin D, a transcriptional inhibitor. The expression level of claudin-2 mRNA was time-dependently decreased in the presence of actinomycin D (Figure 5). Quercetin significantly enhanced the decrease in claudin-2 expression. These results indicate that quercetin decreases the stability of claudin-2 mRNA.

### 3.5. Effects of Polyadenylation and miRNA on the Stability of Claudin-2 mRNA

**Figure 6:**
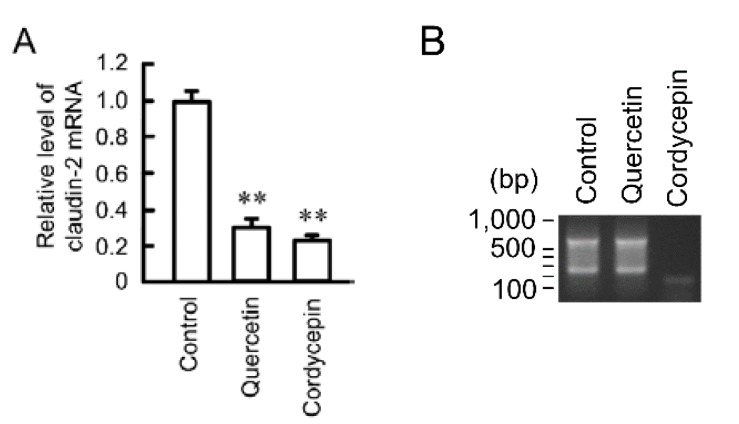
Effect of quercetin on polyadenylation. (**A**) A549 cells were incubated in the absence (control) and presence of 50 μM quercetin or 10 μM cordycepin for 6 h. The expression level of mRNA was quantified by real-time PCR using primers for claudin-2 and β-actin, and was represented relative to values at control; (**B**) After reverse transcription using Olig-dT-3 sites adaptor primer, 3′-RACE PCR was carried out. The PCR products were visualized with ethidium bromide. *n* = 3–4. ** *p* < 0.01 is significantly different from control.

The stability of mRNAs is regulated by polyadenylation and mRNAs with long poly (A) are more stable than those with short poly (A) [32]. We examined the effect of cordycepin, an inhibitor of polyadenylation, on the expression level of claudin-2 mRNA. Both quercetin and cordycepin decreased the expression level of claudin-2 mRNA (Figure 6A). In the same samples, 3′-UTR of claudin-2 is amplified by PCR reaction in control and quercetin-treated cells (Figure 6B). In contrast, the short and faint band was detected in the cordycepin-treated cells.

### 3.6. Rescue of Quercetin-Induced Decrease in Claudin-2 Expression by miR-16 Inhibitor

**Figure 7:**
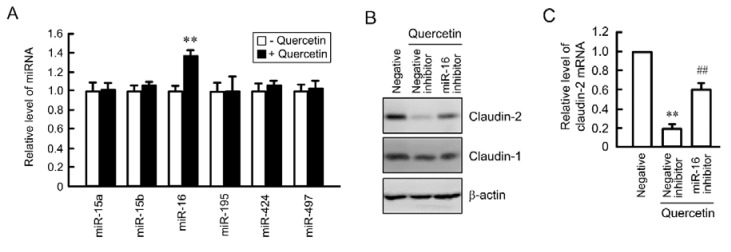
Inhibition of quercetin-induced decrease in claudin-2 expression by miR-16 inhibitor. (**A**) A549 cells were incubated in the absence and presence of 50 μM quercetin for 24 h. The expression level of miRNA was quantified by real-time PCR using primers for miR-15a, 15b, 16, 195, 424, and 497, and was represented relative to values at control; (**B** and **C**) Cells were transfected with 40 nM negative or 40 nM miR-16 inhibitor. After 24 h of transfection, the cells were incubated in the absence and presence of 50 μM quercetin for 24 h. The expression level of mRNA was quantified by real-time PCR using primers for claudin-2 and β-actin, and was represented relative to values at control. *n* = 3–4. ** and ^##^ *p* < 0.01 significantly different from negative control and negative control with quercetin, respectively.

miRNA is also involved in the regulation of mRNA stability [17,18]. TargetScan analysis indicated that the 3′-UTR of claudin-2 contains putative binding sites for miR-15a, 15b, 16, 195, 424, and 497. Quercetin increased miR-16 expression without affecting the expression levels of miR-15a, 15b, 195, 424, and 497 (Figure 7A). The expression levels of claudin-2 mRNA and protein were decreased by quercetin, which were significantly inhibited by miR-16 inhibitor (Figure 7B,C).

## 4. Discussion

The elevation of claudin-2 expression is reported in cancer tissues including lung [27], liver [33], colon [34], and stomach [35]. We recently found that claudin-2 up-regulates the expression of cell cycle regulators such as ZO-1 associated nucleic acid binding protein and cyclin D1 in the nuclei of A549 cells, resulting in the enhancement of proliferation [16]. Similarly, forced claudin-2 expression in colon cancer cells increases tumor growth in mice [34]. Therefore, claudin-2 may be not only a novel biomarker but also a therapeutic target of cancer. Claudin-2 is expressed in several normal tissues including the small intestine and renal proximal tubule. The transcriptional activity of claudin-2 is up-regulated by STAT3 [29], GATA4 [30], and cdx1, cdx2 and hepatocyte nuclear factor-1α [31]. We reported that the EGFR/MEK/ERK/c-Fos pathway up-regulates claudin-2 expression in A549 cells [27]. However, other regulatory mechanism of claudin-2 expression has not been fully investigated.

The effects of flavonoids on the function of TJs and the expression of claudins have been examined in epithelial cells. Quercetin enhances intestinal barrier in Caco-2 cells through the elevation of claudin-4 [36] and through the assembly of zonula occludens-2, occludin, and claudin-1 and the expression of claudin-4 [37]. Genistein and daidzein restore lipopolysaccharide-induced impairment of barrier function in endometrial cells [38]. Here, we found that quercetin decreases claudin-2 expression mediated via up-regulating the instability of claudin-2 mRNA in A549 cells. The expression of claduin-4 is not detected in A549 cells [28] and was not changed by quercetin (data not shown). U0126 and LY-294002 inhibited the phosphorylation of ERK1/2 and Akt, respectively, and both of them decreased claudin-2 expression. However, quercetin did not inhibit the phosphorylation of ERK1/2 and Akt, whereas it decreased claudin-2 expression. These results suggest that quercetin decreases claudin-2 expression mediated by the different mechanisms of U0126 and LY-294002. So far, claudin-2 has been reported to regulate paracellular permeability and remodeling [39]. In contrast, quercetin did not change barrier functions including transepithelial electrical resistance and the permeability to dextran (Supplementary Figure S2), although it decreased claudin-2 expression under our experimental conditions. We suggest that the inconsistency may be attributed to the difference of cell density in the cultures.

The stability of mRNA varies range from about 15 min to more than 24 h [40]. The half-life of mRNAs is regulated by *cis*-elements of mRNA and *trans*-acting RNA binding proteins. Human antigen R (HuR), an mRNA-binding protein, increases the stability of several target mRNAs. The HuR-mediated stabilization of mRNA is involved in carcinogenesis and onset of inflammatory disease and Alzheimer’s disease. Quercetin inhibits the interaction of HuR to the AU-rich element (ARE) of 3′-UTR of tumor necrosis factor-α mRNA in mouse macrophage RAW264.7 cells [41] and myeloid cell leukemia-1 mRNA in human myelomonocytic U-937 cells [42]. The binding of HuR to ARE of the target genes may prevent degradation of mRNA caused by nucleases. In contrast, 3′-UTR of claudin-2 does not contain ARE, suggesting that the stability of claudin-2 mRNA is not regulated by HuR. Polyadenylation is fundamental for mRNA stability, extranuclear transport and translation during development [43]. The majority of genes generate multiple mRNAs as a result of alternative polyadenylation in the 3′-UTR. In 3′-RACE PCR reaction, cordycepin did not amplify DNA of claudin-2, but quercetin amplifies it similar to that in control. We suggest that the inhibition of polyadenylation is not involved in the quercetin-induced instability of claudin-2 mRNA.

The roles of miRNAs have been investigated in normal and tumor lung. Some regulators including Dicer, DGCR8, and Drosha have been reported to be involved in the disruption of miRNAs [44,45]. Knockout mice lacking a key processing enzyme, Dicer, exhibit a failure of epithelial branching [46]. miRNA microarray studies showed that various miRNAs are aberrantly expressed in lung cancer [19]. The expression of miR-16 is down-regulated in 82% of adenocarcinomas and in six NSCLC cell lines [47]. Ectopic expression of miR-16 inhibits cell proliferation, colony formation, migration, and invasion in NSCLC cells [20]. miR-16 induces cell cycle arrest through targeting G1 cyclin, an essential regulator of G1 phase progression [48]. Our data indicate that quercetin decreases claudin-2 expression mediated by the elevation of miR-16 expression. The correlation of claudins and miRNA is reported in other tissue cells. Over expression of miR-155 prevents tumorigenesis in human ovarian cancer mediated by down-regulation of claudin-1 [24]. Down-regulation of miR-1303 prevents proliferation, migration, and invasion of gastric cancer cells mediated by up-regulation of claudin-18 [26]. Proliferation of A549 cells is inhibited by quercetin [5] and the knockdown of claudin-2 [16]. We suggest that quercetin prevents tumorigenesis in human lung cancer partially mediated via the elevation of miR-16 and decrease in claudin-2 expression. Quercetin-rich diet affects the expression of miRNA and is associated with lower risk of lung cancer. We need further study to clarify whether quercetin decreases claudin-2 expression mediated by the up-regulation of miR-16 using *in vivo* model system.

## 5. Conclusions

In the present study, we found that quercetin decreases claudin-2 expression in A549 cells. Quercetin decreased the stability of claudin-2 mRNA without inhibiting its promoter activity. The expression of miR-16 was increased by quercetin, but that of miR-15a, 15b. 195, 424, and 497 was not. Knockdown of miR-16 rescued quercetin-induced decrease in claudin-2. These results indicate that quercetin is a physiologically active substance of foods which decreases claudin-2 expression in A549 cells. Claudin-2 and miR-16 may be potential therapeutic targets for the treatment of adenocarcinomas.

## Supplementary Files

Supplementary File 1

## References

[1] Middleton E., Kandaswami C., Theoharides T.C. (2000). The effects of plant flavonoids on mammalian cells: Implications for inflammation, heart disease, and cancer. Pharmacol. Rev..

[2] Yoshida M., Sakai T., Hosokawa N., Marui N., Matsumoto K., Fujioka A., Nishino H., Aoike A. (1990). The effect of quercetin on cell cycle progression and growth of human gastric cancer cells. FEBS Lett..

[3] Choi J.A., Kim J.Y., Lee J.Y., Kang C.M., Kwon H.J., Yoo Y.D., Kim T.W., Lee Y.S., Lee S.J. (2001). Induction of cell cycle arrest and apoptosis in human breast cancer cells by quercetin. Int. J. Oncol..

[4] Vijayababu M.R., Kanagaraj P., Arunkumar A., Ilangovan R., Aruldhas M.M., Arunakaran J. (2005). Quercetin-induced growth inhibition and cell death in prostatic carcinoma cells (PC-3) are associated with increase in p21 and hypophosphorylated retinoblastoma proteins expression. J. Cancer Res. Clin. Oncol..

[5] Nguyen T.T., Tran E., Nguyen T.H., Do P.T., Huynh T.H., Huynh H. (2004). The role of activated MEK-ERK pathway in quercetin-induced growth inhibition and apoptosis in A549 lung cancer cells. Carcinogenesis.

[6] Zhang J.Y., Yi T., Liu J., Zhao Z.Z., Chen H.B. (2013). Quercetin induces apoptosis via the mitochondrial pathway in KB and KBv200 cells. J. Agric. Food Chem..

[7] Yang J.H., Hsia T.C., Kuo H.M., Chao P.D., Chou C.C., Wei Y.H., Chung J.G. (2006). Inhibition of lung cancer cell growth by quercetin glucuronides via G2/M arrest and induction of apoptosis. Drug Metab. Dispos..

[8] Tsukita S., Yamazaki Y., Katsuno T., Tamura A. (2008). Tight junction-based epithelial microenvironment and cell proliferation. Oncogene.

[9] Powell D.W. (1981). Barrier function of epithelia. Am. J. Physiol. Gastrointest. Liver Physiol..

[10] Matter K., Balda M.S. (2003). Signalling to and from tight junctions. Nat. Rev. Mol. Cell Biol..

[11] Mineta K., Yamamoto Y., Yamazaki Y., Tanaka H., Tada Y., Saito K., Tamura A., Igarashi M., Endo T., Takeuchi K. (2011). Predicted expansion of the claudin multigene family. FEBS Lett..

[12] Turksen K., Troy T.C. (2004). Barriers built on claudins. J. Cell Sci..

[13] Ding L., Lu Z., Lu Q., Chen Y.H. (2013). The claudin family of proteins in human malignancy: A clinical perspective. Cancer Manag. Res..

[14] Chao Y.C., Pan S.H., Yang S.C., Yu S.L., Che T.F., Lin C.W., Tsai M.S., Chang G.C., Wu C.H., Wu Y.Y. (2009). Claudin-1 is a metastasis suppressor and correlates with clinical outcome in lung adenocarcinoma. Am. J. Respir. Crit. Care Med..

[15] Lu Z., Ding L., Hong H., Hoggard J., Lu Q., Chen Y.H. (2011). Claudin-7 inhibits human lung cancer cell migration and invasion through ERK/MAPK signaling pathway. Exp. Cell Res..

[16] Ikari A., Watanabe R., Sato T., Taga S., Shimobaba S., Yamaguchi M., Yamazaki Y., Endo S., Matsunaga T., Sugatani J. (2014). Nuclear distribution of claudin-2 increases cell proliferation in human lung adenocarcinoma cells. Biochim. Biophys. Acta.

[17] Jansson M.D., Lund A.H. (2012). Microrna and cancer. Mol. Oncol..

[18] Fabian M.R., Sonenberg N., Filipowicz W. (2010). Regulation of mRNA translation and stability by microRNAs. Annu. Rev. Biochem..

[19] Mallick R., Patnaik S.K., Yendamuri S. (2010). MicroRNAs and lung cancer: Biology and applications in diagnosis and prognosis. J. Carcinog..

[20] Ke Y., Zhao W., Xiong J., Cao R. (2013). Downregulation of miR-16 promotes growth and motility by targeting HDGF in non-small cell lung cancer cells. FEBS Lett..

[21] Wang Q., Li X., Zhu Y., Yang P. (2014). MicroRNA-16 suppresses epithelial-mesenchymal transitionrelated gene expression in human glioma. Mol. Med. Rep..

[22] Cimmino A., Calin G.A., Fabbri M., Iorio M.V., Ferracin M., Shimizu M., Wojcik S.E., Aqeilan R.I., Zupo S., Dono M. (2005). miR-15 and miR-16 induce apoptosis by targeting BCL2. Proc. Natl. Acad. Sci. USA.

[23] Zhang G.J., Xiao H.X., Tian H.P., Liu Z.L., Xia S.S., Zhou T. (2013). Upregulation of microRNA-155 promotes the migration and invasion of colorectal cancer cells through the regulation of claudin-1 expression. Int. J. Mol. Med..

[24] Qin W., Ren Q., Liu T., Huang Y., Wang J. (2013). MicroRNA-155 is a novel suppressor of ovarian cancer-initiating cells that targets CLDN1. FEBS Lett..

[25] Elfimova N., Sievers E., Eischeid H., Kwiecinski M., Noetel A., Hunt H., Becker D., Frommolt P., Quasdorff M., Steffen H.M. (2013). Control of mitogenic and motogenic pathways by miR-198, diminishing hepatoma cell growth and migration. Biochim. Biophys. Acta.

[26] Zhang S.J., Feng J.F., Wang L., Guo W., Du Y.W., Ming L., Zhao G.Q. (2014). miR-1303 targets claudin-18 gene to modulate proliferation and invasion of gastric cancer cells. Dig. Dis. Sci..

[27] Ikari A., Sato T., Watanabe R., Yamazaki Y., Sugatani J. (2012). Increase in claudin-2 expression by an EGFR/MEK/ERK/c-Fos pathway in lung adenocarcinoma A549 cells. Biochim. Biophys. Acta.

[28] Ikari A., Sato T., Takiguchi A., Atomi K., Yamazaki Y., Sugatani J. (2011). Claudin-2 knockdown decreases matrix metalloproteinase-9 activity and cell migration via suppression of nuclear Sp1 in A549 cells. Life Sci..

[29] Garcia-Hernandez V., Flores-Maldonado C., Rincon-Heredia R., Verdejo-Torres O., Bonilla-Delgado J., Meneses-Morales I., Gariglio P., Contreras R.G. (2015). EGF regulates claudin-2 and -4 expression through SRC and STAT3 in MDCK cells. J. Cell. Physiol..

[30] Escaffit F., Boudreau F., Beaulieu J.F. (2005). Differential expression of claudin-2 along the human intestine: Implication of GATA-4 in the maintenance of claudin-2 in differentiating cells. J. Cell. Physiol..

[31] Sakaguchi T., Gu X., Golden H.M., Suh E., Rhoads D.B., Reinecker H.C. (2002). Cloning of the human claudin-2 5′-flanking region revealed a TATA-less promoter with conserved binding sites in mouse and human for caudal-related homeodomain proteins and hepatocyte nuclear factor-1alpha. J. Biol. Chem..

[32] Guhaniyogi J., Brewer G. (2001). Regulation of mrna stability in mammalian cells. Gene.

[33] Halasz J., Holczbauer A., Paska C., Kovacs M., Benyo G., Verebely T., Schaff Z., Kiss A. (2006). Claudin-1 and claudin-2 differentiate fetal and embryonal components in human hepatoblastoma. Hum. Pathol..

[34] Dhawan P., Ahmad R., Chaturvedi R., Smith J.J., Midha R., Mittal M.K., Krishnan M., Chen X., Eschrich S., Yeatman T.J. (2011). Claudin-2 expression increases tumorigenicity of colon cancer cells: Role of epidermal growth factor receptor activation. Oncogene.

[35] Xin S., Huixin C., Benchang S., Aiping B., Jinhui W., Xiaoyan L., Yu W.B., Minhu C. (2007). Expression of CDX2 and claudin-2 in the multistage tissue of gastric carcinogenesis. Oncology.

[36] Amasheh M., Andres S., Amasheh S., Fromm M., Schulzke J.D. (2009). Barrier effects of nutritional factors. Ann. N. Y. Acad. Sci..

[37] Suzuki T., Hara H. (2009). Quercetin enhances intestinal barrier function through the assembly of zonula [corrected] occludens-2, occludin, and claudin-1 and the expression of claudin-4 in Caco-2 cells. J. Nutr..

[38] Kiatprasert P., Deachapunya C., Benjanirat C., Poonyachoti S. (2015). Soy isoflavones improves endometrial barrier through tight junction gene expression. Reproduction.

[39] Peter Y., Comellas A., Levantini E., Ingenito E.P., Shapiro S.D. (2009). Epidermal growth factor receptor and claudin-2 participate in A549 permeability and remodeling: Implications for non-small cell lung cancer tumor colonization. Mol. Carcinog..

[40] Hollams E.M., Giles K.M., Thomson A.M., Leedman P.J. (2002). mRNA stability and the control of gene expression: Implications for human disease. Neurochem. Res..

[41] Chae M.J., Sung H.Y., Kim E.H., Lee M., Kwak H., Chae C.H., Kim S., Park W.Y. (2009). Chemical inhibitors destabilize HuR binding to the AU-rich element of TNF-alpha mRNA. Exp. Mol. Med..

[42] Spagnuolo C., Cerella C., Russo M., Chateauvieux S., Diederich M., Russo G.L. (2011). Quercetin downregulates Mcl-1 by acting on mRNA stability and protein degradation. Br. J. Cancer.

[43] Curinha A., Oliveira Braz S., Pereira-Castro I., Cruz A., Moreira A. (2014). Implications of polyadenylation in health and disease. Nucleus.

[44] Lin P.Y., Yu S.L., Yang P.C. (2010). MicroRNA in lung cancer. Br. J. Cancer.

[45] Ke Y., Zhao W., Xiong J., Cao R. (2013). miR-149 inhibits non-small-cell lung cancer cells EMT by targeting FOXM1. Biochem. Res. Int..

[46] Harris K.S., Zhang Z., McManus M.T., Harfe B.D., Sun X. (2006). Dicer function is essential for lung epithelium morphogenesis. Proc. Natl. Acad. Sci. USA.

[47] Bandi N., Zbinden S., Gugger M., Arnold M., Kocher V., Hasan L., Kappeler A., Brunner T., Vassella E. (2009). miR-15a and miR-16 are implicated in cell cycle regulation in a Rb-dependent manner and are frequently deleted or down-regulated in non-small cell lung cancer. Cancer Res..

[48] Liu Q., Fu H., Sun F., Zhang H., Tie Y., Zhu J., Xing R., Sun Z., Zheng X. (2008). miR-16 family induces cell cycle arrest by regulating multiple cell cycle genes. Nucleic Acids Res..

