# Supplementary material for: Quercetin Decreases Claudin-2 Expression Mediated by Up-Regulation of microRNA miR-16 in Lung Adenocarcinoma A549 Cells
Source: Nutrients. 2015 Jun 8;7(6):4578–92. doi: 10.3390/nu7064578 (PMC4488803; doi:10.3390/nu7064578)
Supplement: Supplementary File 1 [file nutrients-07-04578-s001.docx]

Supplementary Materials


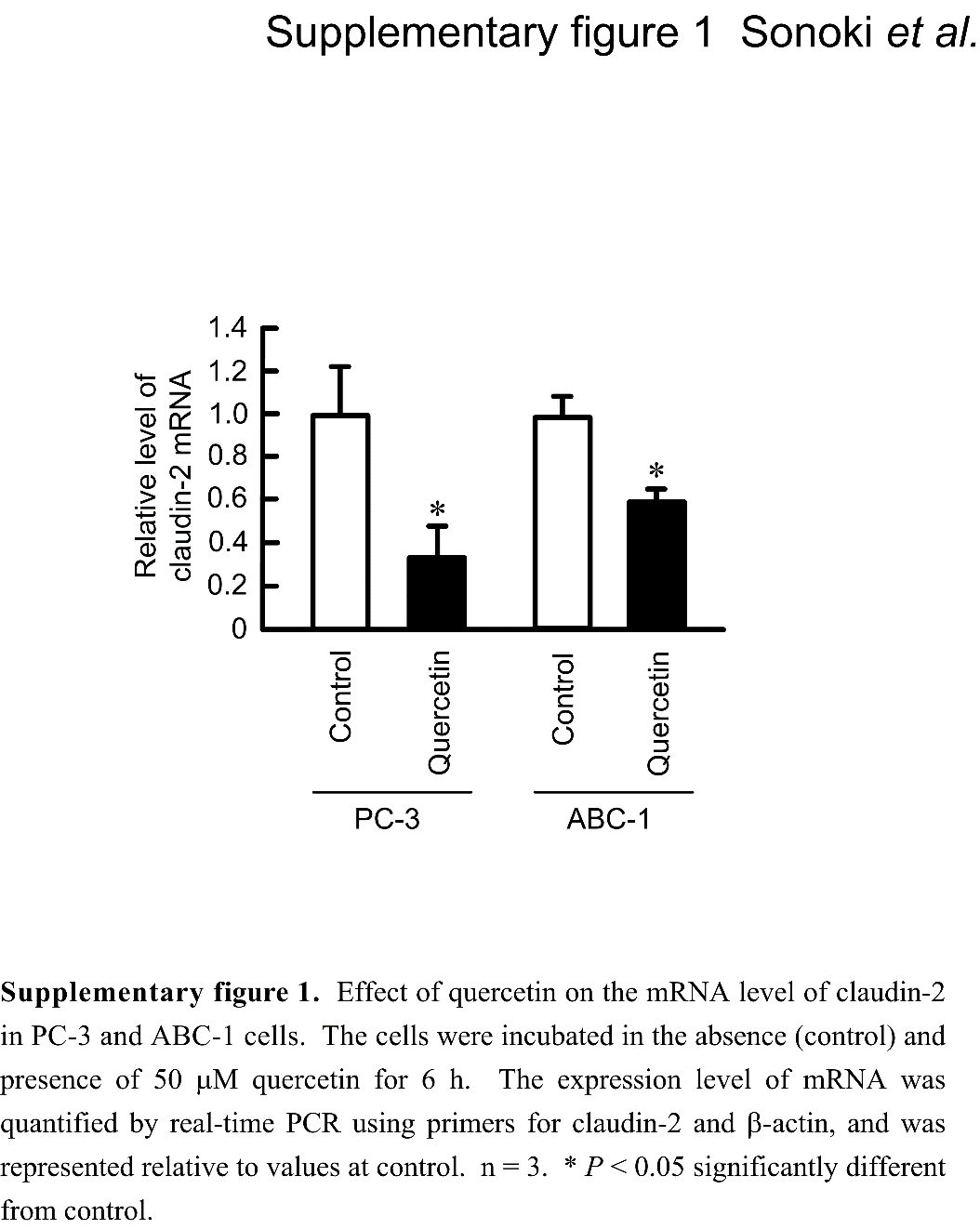


**Figure S1.** Effect of quercetin on the mRNA level of claudin-2 in PC-3 and ABC-1 cells. The cells were incubated in the absence (control) and presence of 50 μM quercetin for 6 h. The expression level of mRNA was quantified by real-time PCR using primers for claudin-2 and β-actin, and was represented relative to values at control. *n* = 3. * *p* < 0.05 significantly different from control.


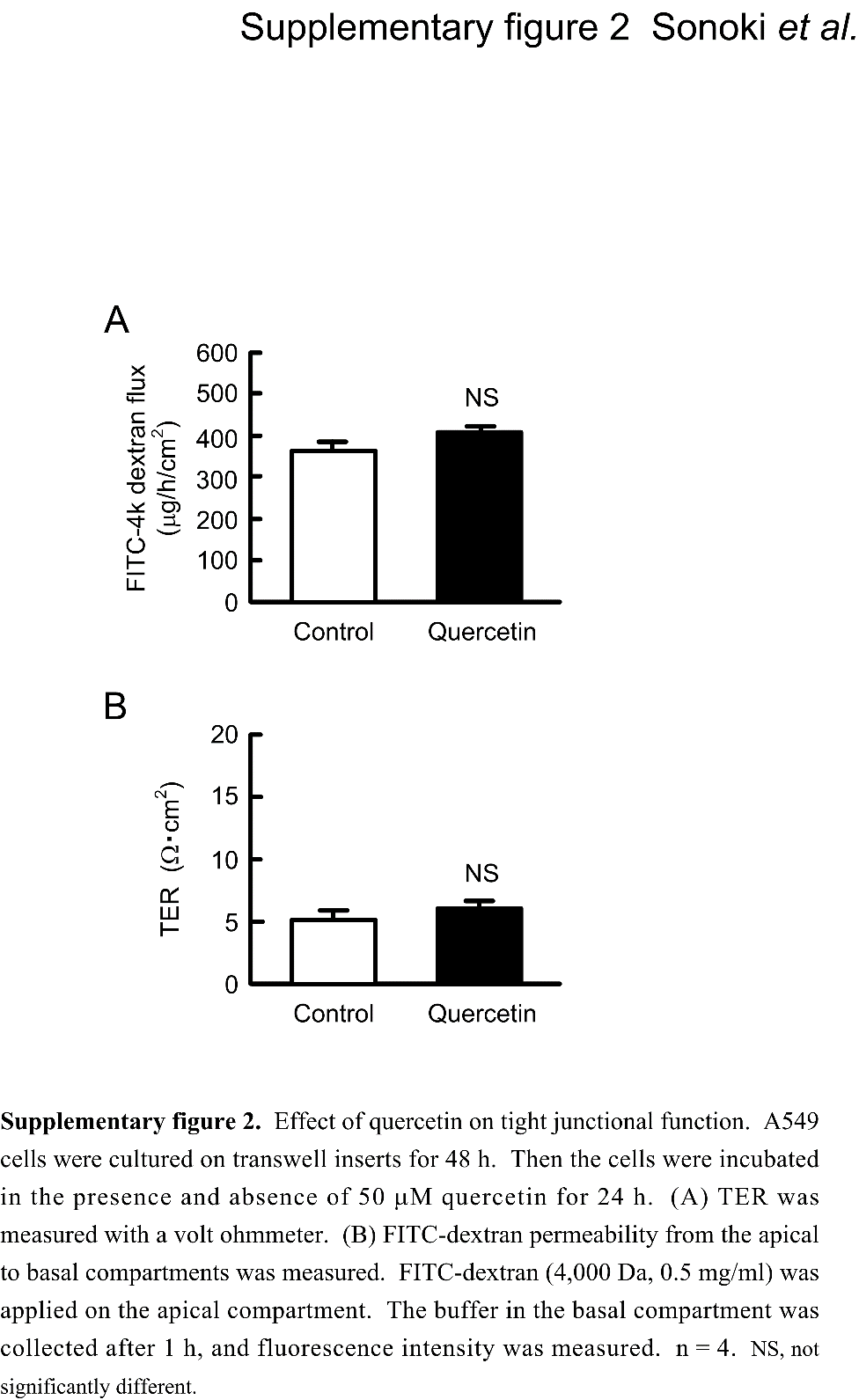


**Figure S2.** Effect of quercetin on tight junctional function. A549 cells were cultured on transwell inserts for 48 h. Then the cells were incubated in the presence and absence of 50 μM quercetin for 24 h. (**A**) TER was measured with a volt ohmmeter; (**B**) FITC-dextran permeability from the apical to basal compartments was measured. FITC-dextran (4000 Da, 0.5 mg/mL) was applied on the apical compartment. The buffer in the basal compartment was collected after 1 h, and fluorescence intensify was measured. *n* = 4, NS, not significantly different.

**Table S1.** Primers for PCR amplification.

| **Gene** | **Direction** | **Sequence** |
| --- | --- | --- |
| Claudin-1 | Forward | 5′-ATGAGGATGGCTGTCATTGG-3′ |
| Claudin-1 | Reverse | 5′-ATTGACTGGGGTCATAGGGT-3′ |
| Claudin-2 | Forward | 5′-ATTGTGACAGCAGTTGGCTT-3′ |
| Claudin-2 | Reverse | 5′-CTATAGATGTCACACTGGGTGATG-3′ |
| β-Actin | Forward | 5′-CCTGAGGCACTCTTCCAGCCTT-3′ |
| β-Actin | Reverse | 5′-TGCGGATGTCCACGTCACACTTC-3′ |
| miR-15a | Forward | 5′-TAGCAGCACATAATGGTTTGTG-3′ |
| miR-15b | Forward | 5′-TAGCAGCACATCATGGTTTACA-3′ |
| miR-16 | Forward | 5′-TAGCAGCACGTAAATATTGGCG-3′ |
| miR-195 | Forward | 5′-TAGCAGCACAGAAATATTGGC-3′ |
| miR-424 | Forward | 5′-CAGCAGCAATTCATGTTTTGAA-3′ |
| miR-497 | Forward | 5′-CAGCAGCACACTGTGGTTTGT-3′ |

© 2015 by the authors; licensee MDPI, Basel, Switzerland. This article is an open access article distributed under the terms and conditions of the Creative Commons Attribution license (http://creativecommons.org/licenses/by/4.0/).
